# Measurement of angle of progression by trans perineal-ultrasound in labour to predict mode of delivery in nulliparous women

**DOI:** 10.12669/pjms.41.1.9930

**Published:** 2025-01

**Authors:** Humaira Masood, Rubaba Abid, Mohammad Ahmed, Munir Mehmood

**Affiliations:** 1 Humaira Masood Senior Registrar, Department of Obstetrics and Gynaecology, Benazir Bhutto Hospital Rawalpindi Medical University, Rawalpindi, Pakistan; 2 Rubaba Abid Associate Professor, Department of Obstetrics and Gynaecology, Benazir Bhutto Hospital Rawalpindi Medical University, Rawalpindi, Pakistan; 3 Mohammad Ahmed, MBBS Department of Obstetrics and Gynaecology, Benazir Bhutto Hospital Rawalpindi Medical University, Rawalpindi, Pakistan; 4 Munir Mehmood, MBBS Department of Obstetrics and Gynaecology, Benazir Bhutto Hospital Rawalpindi Medical University, Rawalpindi, Pakistan

**Keywords:** Angle of descent, Angle of progression, Trans-perineal ultrasound

## Abstract

**Objective:**

The objective of the study was to assess whether the measurement of the angle of progression in nulliparous women in labour can predict the mode of delivery.

**Methods:**

This prospective observational study was conducted at Benazir Bhutto Hospital, Rawalpindi Medical University from 16^th^ February to 25^th^ March 2024. Nulliparous pregnant women in the active first stage of labour with singleton pregnancy and cephalic presentation were included in the study after taking informed consent. The angle of progression (AOP) was measured by trans-perineal ultrasound using the curvilinear probe. The primary outcome was the mode of delivery in relation to angle of progression.

**Results:**

Seventy-eight patients were included in the study. Angle of progression was wider in patients who underwent vaginal delivery and narrower in patients who underwent cesarean section. AOP of 121 or more is associated with 86.6% of vaginal delivery and an angle of progression of 70 or less is associated with 83.3% of cesarean section.

**Conclusion:**

The angle of progression is a reliable tool for predicting the mode of delivery. Wider angle is associated with vaginal delivery and a narrower angle is associated with cesarean section.

## INTRODUCTION

Prediction of the mode of delivery is important for the woman as it increases satisfaction of the woman and improves her birth experience. Accurate assessment of labour progress and fetal head descent became a challenge for obstetricians. Traditionally station of fetal head is assessed by digital vaginal examination. However, this approach is subjective when there is caput succendum.^1^ It is also painful for the woman.^2^ Intrapartum trans perineal ultrasound has been found as a more objective means to determine fetal head station.^3^ Transperineall ultrasound has been studied as a useful method for monitoring of progress of labour and to predict the mode of delivery.^4^

There are different parameters to measure by transperineal ultrasound,^5^ angle of progression is the one parameter. It is measured between the long axis of the symphysis pubis and a line extending from its inferior edge tangentially to the fetal skull.^6^ This angle can reliably predict the mode of delivery if calculated in labouring women.^7^ Accurate prediction increases women satisfaction and decreases complications during labour.^8^ Angle of progression has emerged as a promising tool in this regard. Research indicates that this technique gives a more objective measurement of fetal head descent as compared to manual examinations.^9^ Importance of this angle has been highlighted in various studies. In our setups there are limited options for pain management due to the non-availability of expertise and cost issues so painful labours ending in cesarean section were very distressing for the women.

Also, caesarean section in the second stage of labour increases maternal and fetal complications as compared to elective cesarean section. Predicting whether the woman will delivered vaginally or by caesarean section in early labour is important to prevent complications and to improve women’s birth experience. This study was conducted to assess the angle of progression in labouring women in the prediction of the mode of delivery in our population. By exploring this approach, we will enhance the quality of obstetric care and provide better outcome for the mother and fetus.

## METHODS

This prospective study was conducted in the obstetrics and gynecology labour ward in a tertiary care hospital. The study was conducted from 16^th^ February to 25^th^ March 2024. Total no of maternities in this hospital in the month of march 2024 were 1119. Total no of caesarean sections was 487(43.53%) and vaginal deliveries were 632(56.47%). Primigravidas were 279(41.7%) Sample size was calculated by using WHO sample size calculator, with the calculation details are confidence level 95%, anticipated population proportion is 72%(elena brunelli et al,2021), absolute precision required is 10%, sample size is 78 cases. The inclusion criteria were nulliparous women in the active first stage of labor from 4cm of cervical dilatation up to 10 cm with a singleton-term pregnancy and cephalic presentation. Exclusion criteria include extremes of estimated fetal weight (>4 kg or <2.5 kg), pregnancy with medical disorders, and preterm labour.

### Ethical Approval:

It was approved by the institutional review board and ethical committee (with reference no 625/IREF/RMU/2024 Dated 15/2/2024).

All the primiparous women who were in active stage of labour were included in the study. Written consent was taken from each participating woman after an explanation of the whole procedure. These women will be assessed for angle of progression by transperineal ultrasound. The ultrasound machine was Honda with model no 2600. The curvilinear probe of 2.8-3.5-5 MHz was used for the image. The ultrasound was performed by the registrar who was FCPS in obstetrics and gynaecology with post FCPS experience of three years or more. The senior registrar is experienced in performing ultrasound, it will increase generalizability of the ultrasound examination. Also the measurement of the angle is between two bony structures and can be measured easily.^10^ Women were asked to void and lie in a dorsal position for the ultrasound.

**Fig.1:**
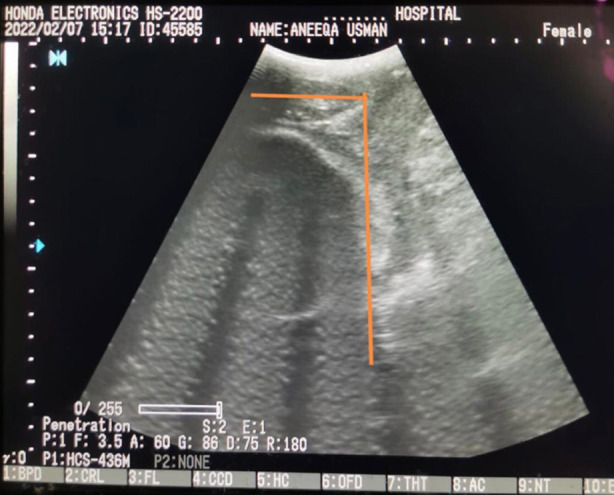
Ultrasound image showing angle of progression.

The curvilinear probe will be enclosed in a latex glove covered with ultrasound gel and then placed between the labia below the pubic symphysis, in the long axis. The image showing the pubic symphysis and fetal skull will be localized and the angle was measured. (Fig.1) The angle of progression is the angle between the longitudinal axis of the pubic bone and a line joining the lowest edge of the pubis to the lowest convexity of the fetal skull.^10^ The labour progress of women will be managed by labour and delivery team according to the departmental protocol. The labour team was blinded by the results of the ultrasound. The primary outcome was the mode of delivery in relation to the angle of progression. The secondary outcome was the cutoff level of the angle for predicting normal vaginal delivery.

### Statistical analysis:

Data was analysed by SPSS version 24. For quantitative variables, means and standard deviations were calculated. For qualitative variables frequency and percentages were calculated. Angle of progression was compared with mode of delivery(svd/LSCS) by using chi square test. The p value of <0.05 was considered statistically significant

## RESULTS

Seventy-eight women were included in the study. Their demographic characteristics are shown in Table-I. Women presented in spontaneous labour were 65(83.3%) and induced labour were 13(16.6%). In 78 patients 44 (56.4%) delivered vaginally and 34(43.6%) underwent caesarean section. Angle of progression was narrower in patients who underwent cesarean section and wider in patients with vaginal delivery.

**Table-I:** Demographic characteristics.

Demographic characteristics	n	Minimum	Maximum	Mean	Std.deviation
Age (years)	78	18.00	37.00	23.16	3.82
Gestational age (weeks)	78	37.00	41.00	38.91	1.17
Baby weight (kg)	78	2.60	3.40	3.09	0.18

The sensitivity at angle 121-140 is 0.866. This means that out of all vaginal deliveries in this angle range, 86.6% were correctly identified when angles were individually analyzed we see women with an angle of progression of 121 or more,86.6% were delivered vaginally. Women with an angle of progression of 70 or less, 83.3% were delivered by caesarean section. A cut-off level of 121 or more is associated with a successful vaginal delivery and an angle of 70 or less is associated with a cesarean section.

**Fig.2:**
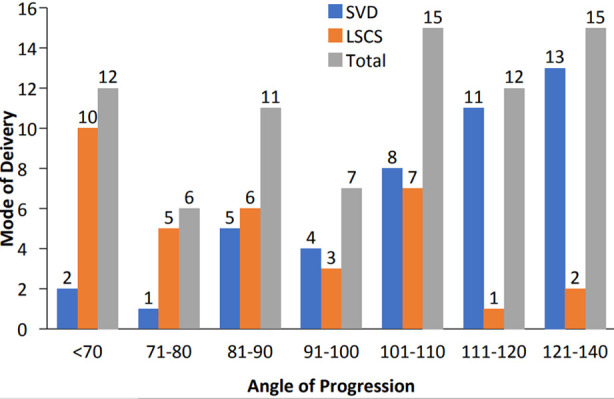
Comparison between different angles of progression with mode of delivery.

The relationship of different angles with the mode of delivery is shown in Table-II and Fig.2. Indications of cesarean sections are shown in Table-III. Overall, the sensitivity suggests that the angle of progression might be a good predictor of vaginal delivery in the 121-140 degree range.

**Table-II:** Angle of progression with mode of delivery.

Angle of progression	SVD	LSCS	Total no of patients	p-value
<70	2(4.5%)	10 (29.4%)	12(15.4%)	.001
71-80	1(2.3%)	5(14.7%)	6(7.7%)	
81-90	5(11.4%)	6 (17.6%)	11(14.1%)	
91-100	4(9.1%)	3(8.8%)	7(9.0%)	
101-110	8(18.2%)	7(20.6%)	15(19.2%)	
111-120	11(25%)	1(2.9%)	12(15.4%)	
121-140	13(29.5%)	2(5.9%)	15(19.2%)	

**Table-III:** Indications of cesarean section.

Indication	Frequency	Percentage
Primary arrest	12	15.4%
Secondary arrest	12	15.4%
Pathological CTG	5	6.4%
Meconium	7	9.0%

## DISCUSSION

Trans-perineal ultrasound has been used in various indications of labour. It has been found a useful tool in labour monitoring. We performed AOP in the first stage of labour and found that angle can reliably predict the mode of delivery. AOP of 121 is associated with 86.6% of vaginal delivery. When measured in the active first stage of labour we found that a wider angle of progression is associated with vaginal delivery and a narrow angle of progression is associated with an increased chance of cesarean section. AOP has been explored in many studies and found to be an objective means of prediction of mode of delivery.^11^ Furthermore the angle was found to be superior to digital examination.^12^

In our study, 86.6% of the woman with angle of progression >121 delivered vaginally. At an angle of progression of 70 or less 83.3% were delivered by cesarean section. A study by Kalachi et al.^13^ documents a strong relationship between the measurement of the angle of progression and the type of delivery. They showed that at an angle of 120, the probability of vaginal delivery is 90%. Also, a study by Malik and singh.^11^ found that an AOP of 116 is predictive of vaginal delivery and angles of <90 are more toward cesarean section. Another study demonstrates similar results that an AOP of 120 was associated with normal vaginal delivery.^14^ These results are similar to our study which shows that a wider angle is associated with successful vaginal delivery.

Several studies have showed the relationship between AOP with the fetal head station. AOP of 120 is associated with station zero.^6^ A magnetic resonance imaging study done in a pregnant woman showed that an AOP of 121 is associated with the fetal head station of zero.^15^ Barbera in his study showed AOP OF 99 corelates with the level of ischial spines.^16^ A study compared AOP with clinical assessment and found AOP of 116 is equal to zero station.^17^ As in our study AOP of 121 is mostly associated with vaginal delivery nevertheless showed that the head is engaged at this angle. Measuring the Angle of Progression (AOP) during labor is a significant factor in reducing maternal and fetal complications associated with prolonged labor and emergency cesarean section. Our study demonstrated a strong correlation between AOP and the mode of delivery. Women with an AOP of 121 were significantly more likely to deliver vaginally, while those with an AOP of 70 or less had a higher probability of requiring cesarean section.

These findings offer valuable insights for counseling nulliparous women considering vaginal delivery versus elective cesarean section. By assessing AOP early in labor, healthcare providers can better anticipate the likelihood of cesarean section and plan accordingly, potentially avoiding unnecessary labor pain and complications. Prediction of the mode of delivery is important for the woman as it improves her birth experience.^18^ Also our women in labour scarcely adopt epidural or other analgesia in labour so after taking labour pains, the decision to undergo a cesarean section after failed progress of labour is distressing for the woman. The complication rate is also high for operations in the second stage of labour. The strength of our study is that labour and delivery team was blinded to the AOP measurement and their management was not affected by the results thus preventing bias.

### Limitations:

The limitation of the study is that we have taken AOP on still ultrasound images. Studies have already shown that there is slight and insignificant inter and intra-observer variation in different sonographers.^19^ The other factor is that the analysis was based on a limited sample size, which may restrict the generalizability of the findings to a broader population also there is a lack of control of certain confounding factors like fetal distress.

## CONCLUSION

Measurement of the angle of progression by using the trans-perineal ultrasound is a useful and easily implemented tool. Wider angle is associated with successful vaginal delivery and a narrower angle is associated with cesarean section. However, due to the small no of subjects, the cut-off value for predicting vaginal delivery and cesarean section cannot be reliably concluded. RCTs are required to predict the cutoff levels reliably.

### Authors’ Contribution:

**HM:** Designed and data collection, manuscript writing, final approval, responsible for the integrity of the study.

**RA:** Did literature search, critical review..

**MA:** Did statistical analysis and manuscript editing.

**MM:** Did literature search, manuscript writing.

All authors have read the final version and are responsible for the integrity of the study.
